# Elevated serum Activin A in chronic obstructive pulmonary disease with skeletal muscle wasting

**DOI:** 10.6061/clinics/2019/e981

**Published:** 2019-06-20

**Authors:** Guanghui Zhou, Xianhua Gui, Ruhua Chen, Xingli Fu, Xiuhai Ji, Hui Ding

**Affiliations:** I Department of Respiratory Medicine, Yixing People Hospital, Affiliated Jiangsu University, 214200, China; II Department of Respiratory Medicine, Nanjing Drum Tower Hospital, Affiliated Hospital of Nanjing University Medical School, 210008, China; III Jiangsu University Health Science Center, Yizheng Road, Zhenjiang, Jiangsu, 212001, China; IV Department of Oncology, Affiliated Taicang Hospital of Traditional Chinese Medicine, Suzhou, 215400, China

**Keywords:** COPD, Skeletal Muscle, Activin A

## Abstract

**OBJECTIVE:**

Muscle wasting contributes to the reduced quality of life and increased mortality in chronic obstructive pulmonary disease (COPD). Muscle atrophy in mice with cachexia was caused by Activin A binding to ActRIIB. The role of circulating Activin A leading to muscle atrophy in COPD remains elusive.

**METHODS:**

In the present study, we evaluated the relationship between serum levels of Activin A and skeletal muscle wasting in COPD patients. The expression levels of serum Activin A were measured in 78 stable COPD patients and in 60 healthy controls via ELISA, which was also used to determine the expression of circulating TNF-α levels. Total skeletal muscle mass (SMM) was calculated according to a validated formula by age and anthropometric measurements. The fat-free mass index (FFMI) was determined as the fat-free mass (FFM) corrected for body surface area.

**RESULTS:**

Compared to the healthy controls, COPD patients had upregulated Activin A expression. The elevated levels of Activin A were correlated with TNF-α expression, while total SMM and FFMI were significantly decreased in COPD patients. Furthermore, serum Activin A expression in COPD patients was negatively associated with both FFMI and BMI.

**CONCLUSION:**

The above results showed an association between increased circulating Activin A in COPD patients and the presence of muscle atrophy. Given our previous knowledge, we speculate that Activin A contributes to skeletal muscle wasting in COPD.

## INTRODUCTION

As a degenerative systemic manifestation, skeletal muscle atrophy contributes to a determinant of mortality independent of airway obstruction in chronic obstructive pulmonary disease (COPD) 1,2. Traditionally, skeletal muscle atrophy as selective depletion of fat-free mass index (FFMI) and body mass index (BMI) disturbs the potential balance of muscle protein synthesis and degradation in 20-40% of COPD patients depending on definition and disease stage, resulting in decreased muscle function and adaptive capacity 3,4. Total skeletal muscle mass (SMM) constitutes more than half of the total body mass. As an important indicator of nutrition and metabolic function, declining SMM is recognized as having adverse effects on health 5,6. It is necessary to investigate the biological factors inducing muscle atrophy in COPD.

The mechanisms of muscle mass atrophy are highly complex. Li et al. found that TNF-α acted to induce skeletal muscle atrophy in vivo and in vitro 7. Recent research has reported the activated role of Activin A (Act A) on muscle mass degradation via binding to Activin receptor-IIB (ActRIIB) in cachectic mice with lung cancer 8. Act A, a superfamily of transforming growth factor-β (TGF-β), was found to be an inducer of muscle degradation in cachectic mice 9. Although Act A is closely associated with inflammation 10, it is unclear whether serum Act A contributes to muscle wasting in COPD.

Based on previous studies, we hypothesized that serum Act A resulted in muscle wasting in COPD with low BMI, FFMI and SMM. In this study, we measured circulating levels of Act A and correlation with serum TNF-α levels, BMI, FFMI and SMM to investigate the association between Act A and muscle atrophy in COPD.

## MATERIALS AND METHODS

### Patients and study design

This study was performed at Yixing Hospital, Affiliated Jiangsu University, and approved by the ethical committee of Jiangsu University. All participants in this study provided written informed consent. The study methodologies conformed to the standards set by the Declaration of Helsinki. A total of 78 patients with stable COPD and 60 aged-matched healthy controls who smoked for more than 10 years were enrolled. COPD was diagnosed based on criteria 11 showing irreversible airflow obstruction via spirometry (forced expiratory volume in 1s (FEV1) <80% predicted value and FEV1/forced vital capacity (FVC) <70%). All COPD patients were in stable condition at the time of study without any exacerbation of disease or systemic corticosteroid administration within 2 months of their participation in this test. Patients with any comorbid condition associated with muscle wasting (i.e., active inflammatory, illnesses, cancer, congestive heart failure, chronic renal failure or denervation) were excluded.

### Pulmonary function and inflammatory testing

According to well-established methods 12, effective pulmonary function tests, including spirometry, lung volume with body plethysmography, and diffusion carbon monoxide diffusion capacity were used in all patients and controls during the initial evaluation. 

As described previously 13, in order to exclude airway inflammation as a disturbing factor, all participants were examined for the fraction of exhaled nitric oxide (FeNO).

### Anthropometric measurements and body composition

As in a previous report 14, SMM was determined by a validated formula using age and anthropometric measurements. The main parameters of this formula were body height and skinfold corrected limb circumferences as skinfold corrected upper-arm girth (CAG), skinfold corrected calf girth (CCG) and skinfold corrected thigh girth (CTG). According to the standardized anatomic locations 14 and the specific methods 15, circumference measurements were carried out on all participants.

We used dual energy X-ray absorptiometry (DEXA) to provide regional measurements of the FFMI and FFM (General Electric Healthcare, Chicago, IL). FFM was measured from the sum of lean mass and bone mineral content. Moreover, the FFMI was derived from FFM normalized to body surface area. Muscle atrophy was defined as an FFMI less than 15 kg/m^2^ in women and 17 kg/m^2^ in men, as reported previously 16,17.

### Serum biomarker measurements

Peripheral venous blood was collected from each participant between 7 am and 8 am after fasting since 9 pm the previous night as standardized conditions. Serum Activin A and TNF-α were measured using enzyme-linked immunoassays (ELISA) kits (R&D Systems, USA) according to the manufacturer's recommendations.

### Statistical analysis

Data for comparison of unpaired parameters between patients and controls were analyzed with Student's *t* test via SigmaStat software as indicated. The relationship between Act A and other parameters was confirmed via linear regression correlation analysis. A *p*-value <0.05 was considered statistically significant. Data are presented as the mean ± s.e.

## RESULTS

### Subject characteristics

Anthropometric characteristics, pulmonary function data and FeNO results are presented in Table 1. Patients with COPD exhibited significant airflow obstruction in line with reduced FEV1 levels and FEV1/FVC ratios as diagnostic criteria of COPD. BMI index attenuation was established in patients with COPD, while there was no significant difference in terms of age and sex compared with controls. In addition, there were 58 cases among all COPD patients with malnutrition according to a BMI of less than 21 kg/m^2^ as previously reported 15,18 and low FFMI. To eliminate the effects of airway inflammation on Act A expression, we used a FeNO value greater than 25 ppb as a criterion to exclude patients. There was no significant difference in the FeNO results between the two groups.

### Muscle characteristics

As described in Table 2, total body SMM with regards to reliable value and its ratio to body weight was reduced in patients with COPD (18.26±2.24 *vs*. 25.33±3.13). Consequently, local SMM, such as CAG, CTG and CCG, was also significantly decreased in COPD patients compared with controls. Additionally, COPD patients showed remarkable loss of muscle mass, such as reductive FFM and FFMI, as well as other anthropometric measurements (16.27±2.06 *vs*. 22.64±2.73).

### Serum expression of Action A and TNF-α levels

**Figure 1:**
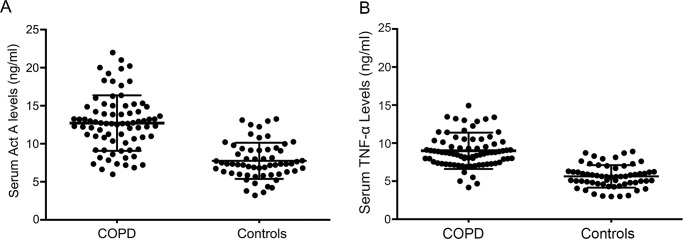
Serum levels of Activin A and TNF-α in COPD patients. A: Elevated serum Activin A levels were present in COPD patients relative to controls. B: Serum TNF-α levels were significantly increased in patients with COPD.

Interestingly, the mean levels of systemic Act A were absolutely elevated in patients with COPD (13.12±3.87 *vs*. 7.28±2.06), whereas serum levels of TNF-α as an important inflammatory factor playing a central role in the pathophysiology in muscle atrophy were also upregulated in COPD patients when compared with controls (9.36±3.18 *vs*. 6.03±1.86) (Figure 1).

### Correlations between serum Act A levels and other parameters

**Figure 2:**
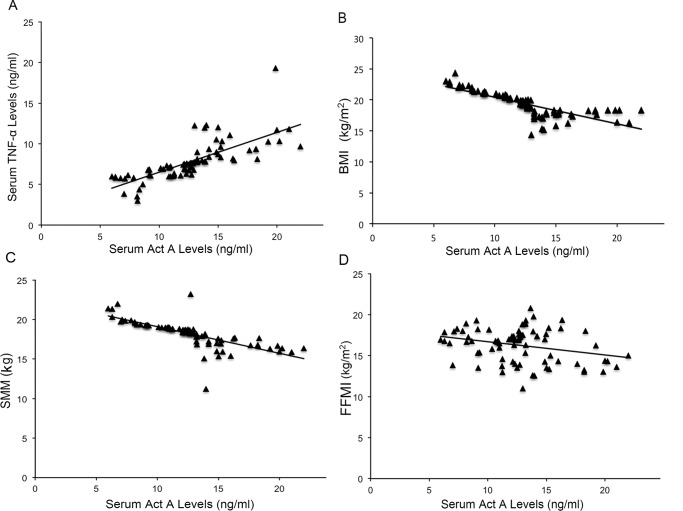
Correlation between Act A levels and indicators of muscle wasting in COPD. A: Positive correlation between Act A and TNF-α levels in COPD patients. B: Negative correlation between Act A and BMI in COPD patients (B) and SMM (C) and FFMI (D).

As shown in Figure 2, regression correlation analysis indicated a significant positive correlation between systemic Act A levels and TNF-α concentrations in patients with COPD (*R*^2^=0.041, *p*=0.025). In addition, serum Act A expression was inversely correlated with BMI in COPD patients (*R*^2^=0.021, *p*=0.004). A negative correlation was also observed between serum Act A and SMM in COPD patients (*R*^2^=0.017, *p*=0.01). In addition, regression correlation analysis showed the inverse correlation between circulation Act A levels and FFMI as an indicator of muscle function (*R*^2^=0.013, *p*=0.008).

## DISCUSSION

The current study reveals that elevated circulating levels of Act A in COPD are associated with muscle atrophy as shown by reductions in BMI, SMM and FFMI. To the best of our knowledge, this is the first report on the relationship between serum Act A and muscle wasting in COPD. Nevertheless, it is necessary to highlight the importance of abnormal serum Act A levels in relation to the muscle mass regulation of COPD patients.

Several previous studies explored that muscle atrophy as a systemic complication of whole body weight is commonly detected in 10%-40% of COPD patients 19. In our study, we also found muscle atrophy in COPD patients showing decreased total-body SMM with regards to its absolute value and its percentage of body weight, as well as BMI. Indeed, the reduced SMM and BMI levels in our data were in line with recent reports of muscle wasting and malnutrition in COPD patients 15,20,21. A previous study reported that the continued loss of protein in muscle mass leads to muscle fiber shrinkage and a reduction in the muscle cross-sectional area (CSA), resulting in marked muscle wasting 22. Some researchers have explored the reduction in FFM and FFMI as malnutrition and muscle atrophy 17 were significant in COPD patients. Interestingly, consistent with these data, we also noticed that there were obvious decreases in FFM and the FFMI in COPD. Although the ECLIPSE group reported elevated BMI and FFMI in a subgroup of COPD patients with persistent systemic inflammation 23, Barker et al. showed that FFM and FFMI levels, such as nutritional depletion and progressive muscle atrophy, were not related to airway inflammation or bacterial colonization 17,24. Furthermore, to remove the impact of systemic and airway inflammation on muscle atrophy, we excluded not only patients with persistent oral glucocorticoid use in our research study but also patients with serum CRP and FeNO levels in COPD and controls. Our data showed a marked reduction in BMI, SMM and FFMI as muscle atrophy in COPD patients but not in healthy controls.

In addition to abnormal conditions, such as denervation, fasting, disuse, oxidative stress and hypoxia 25-27, a number of factors, such as myostatin and Act A, are responsible for muscle weakness due to protein degradation and intrinsic metabolic abnormalities 28,29. Indeed, as a major subfamily of TGF-β 30, Act A has a wide range of biological effects on wound healing, cell proliferation and differentiation, immune response and angiogenesis 31,32. Upregulated Act A has been shown in many catabolic disease states, including ovarian cancer, endometrial adenocarcinoma, prostate, multiple myeloma, esophageal, breast and pancreatic cancers, and noncancer settings, such as chronic renal failure, heart failure, and pulmonary hypertension 33. Most intriguingly, Act A maintenance at high levels in the serum and tumor tissue of cachectic cancer patients suggested its involvement in the pathogenesis of muscle wasting 10,33. Beyond its classic roles, Act A was established as a paracrine/autocrine factor in non-gonadal tissues, including skeletal and heart muscles. Mice treated with Act A experienced an approximately 30% decrease in muscle mass 8. In addition, inhibition of Act A expression by its antagonist follistatin prevented skeletal muscle from insistent degradation. Moreover, recent evidence suggested that Activin A activated signaling in the development and progression of muscle wasting via the ActRIIB, which may be regarded as a therapeutic target for cachexia 8. In line with the observation showing the upregulated expression of Act A in the airway epithelium, airway smooth muscle and alveolar macrophages of COPD patients 10,34, we found increased circulating levels of Act A in COPD patients. Furthermore, to the best of our knowledge, we established the first negative correlations between elevated serum levels of Act A and muscle mass index as BMI, SMM and FFMI in our study, which indicated the contributing role of Act A in muscle degradation in COPD. Together with previous evidence of increased Act A levels with muscle atrophy, our findings indicated a closing linkage of serum Act A levels with skeletal muscle wasting and cachexia in COPD.

Some previous reports indicated the relationship of muscle atrophy with elevated TNF-α (previously named cachectin) due to its tissue depletion and body weight loss abilities 35. Consistent with the results showing elevated TNF-α mRNA levels, which play a key role in the pathophysiology of COPD 36, our study also described increased serum TNF-α levels in patients compared with controls. Indeed, TNF-α was involved in the development of skeletal muscle degradation resulting in loss of muscle mass and function 37. Further studies found that elevated TNF-α in COPD was inversely correlated with SMM and BMI 15. In our study, we further investigated the relationship between Act A and TNF-α to understand the mechanisms of muscle atrophy more clearly. We found a positive correlation between circulating Act A and TNF-α in COPD. Together with the literature, we suggest that increasing serum Act A levels might be stimulated by TNF-α, leading to more significant skeletal muscle wasting in COPD.

Our data first demonstrated that circulating Act A was markedly increased in COPD patients. Muscle wasting in COPD was positively related to elevated Act A and TNF-α but negatively correlated with BMI, FFMI and SMM. We speculated that elevated serum Act A might contribute to muscle atrophy and weight loss in COPD patients.

Our major limitation in this study was the small population of COPD patients, especially when stratified by gender. Future studies will focus on the mechanisms of Act A-induced muscle wasting in COPD. Nevertheless, although limited, our data are sufficient to support the importance of Act A in COPD patients.

## AUTHOR CONTRIBUTIONS

Zhou G, Gui X and Fu X prepared the samples and carried out the data analysis. Chen R and Ji X designed the project. Ding H wrote the manuscript.

## Figures and Tables

**Table 1:** General characteristics of the enrolled participants.

	COPD (N=78)	Control (N=60)	*p*-value
Sex, M (%)	52 (66.7)	35 (58.3)	0.31
Age, (yrs)	67.12±8.37	61.92±6.58	0.26
BMI (kg/m^2^)	19.25±3.25	23.31±4.41	<0.01
FeNO (ppb)	22.16±5.73	20.26±7.72	0.2
FEV_1_ pred (%)	53.66±5.23	89.18±6.18	<0.01
FEV_1_/FVC (%)	47.73±8.22	88.78±4.39	<0.01
RV/TLC (%)	33.45±4.86	29.11±3.65	0.38
DLco (ml/m/mmHg)	24.78±7.42	28.35±8.46	0.35
PH	7.41±0.14	7.38±0.22	0.36
PaO_2_ (mmHg)	78.29±10.76	82.71±13.41	0.27
PaCO_2_ (mmHg)	33.71±9.15	39.26±10.47	0.25
Blood white cell count (10^9^/L)	8.43±0.23	8.83±0.47	0.44
Blood neutrophil (10^9^/L)	5.77±0.39	4.52±0.32	0.38
Blood eosinophil (10^9^/L)	0.46±0.08	0.42±0.11	0.42
CRP (mg/L)	3.23±1.28	4.26±1.14	0.32

BMI, body mass index; FeNO, fraction of exhaled nitric oxide; FEV1, forced expiratory volume at 1s; FVC, force vital capacity; PO_2_ oxygen partial pressure; PCO_2_ carbon dioxide partial pressure; RV, residual volume; TLC, total lung capacity; DLco, total lung carbon monoxide transfer factor; CRP, C reactive protein.

**Table 2:** Muscle characteristics in COPD patients and controls.

	COPD (N=78)	Control (N=60)	*p*-value
CAG (cm)	17.31±2.15	23.65±2.78	<0.01
CTG (cm)	31.63±4.11	44.82±3.09	<0.01
CCG (cm)	24.07±2.17	29.38±2.84	<0.01
SMM (kg)	18.26±2.24	25.33±3.13	<0.01
SMM/Wt (%)	28.85±3.99	40.27±3.77	<0.01
FFM (kg)	41.28±3.91	53.27±2.28	<0.01
FFMI (kg/m^2^)	16.27±2.06	22.64±2.73	<0.01

CAG, skinfold corrected upper-arm girth; CTC skinfold corrected thigh girth; CCG, skinfold corrected calf girth; SMM, total body skeletal mass; Wt, weight; FFM, fat-free mass; FFMI, fat-free mass index.
